# Elevated extracellular calcium ions accelerate the proliferation and migration of HepG2 cells and decrease cisplatin sensitivity

**DOI:** 10.7555/JBR.37.20230067

**Published:** 2023-09-10

**Authors:** Haozhe Xu, Yiming Zhou, Jing Guo, Tao Ling, Yujie Xu, Ting Zhao, Chuanxin Shi, Zhongping Su, Qiang You

**Affiliations:** 1 Department of Geriatrics, Medical Center for Digestive Diseases, the Second Affiliated Hospital of Nanjing Medical University, Nanjing, Jiangsu 210011, China; 2 Affiliated Cancer Hospital & Institute, Guangzhou Medical University, Guangzhou, Guangdong 510095, China; 3 Department of Medical Oncology, Fudan University Shanghai Cancer Center, Shanghai 200032, China; 4 Division of General Surgery, the Second Affiliated Hospital of Nanjing Medical University, Nanjing, Jiangsu 210011, China; 5 Department of Geriatric Gastroenterology, the First Affiliated Hospital of Nanjing Medical University, Institute of Neuroendocrine Tumor, Nanjing Medical University, Nanjing, Jiangsu 210029, China

**Keywords:** HepG2, hepatoblastoma, calcium ion, FAK, CaMKⅡ, cisplatin resistance

## Abstract

Hepatoblastoma is the most frequent liver malignancy in children. HepG2 has been discovered as a hepatoblastoma-derived cell line and tends to form clumps in culture. Intriguingly, we observed that the addition of calcium ions reduced cell clumping and disassociated HepG2 cells. The calcium signal is in connection with a series of processes critical in the tumorigenesis. Here, we demonstrated that extracellular calcium ions induced morphological changes and enhanced the epithelial-mesenchymal transition in HepG2 cells. Mechanistically, calcium ions promoted HepG2 proliferation and migration by up-regulating the phosphorylation levels of focal adhesion kinase (FAK), protein kinase B, and p38 mitogen-activated protein kinase. The inhibitor of FAK or Ca ^2+^/calmodulin-dependent kinase Ⅱ (CaMKⅡ) reversed the Ca ^2+^-induced effects on HepG2 cells, including cell proliferation and migration, epithelial-mesenchymal transition protein expression levels, and phosphorylation levels of FAK and protein kinase B. Moreover, calcium ions decreased HepG2 cells' sensitivity to cisplatin. Furthermore, we found that the expression levels of *FAK* and *CaMKⅡ* were increased in hepatoblastoma. The group with high expression levels of *FAK* and *CaMKⅡ* exhibited significantly lower ImmunoScore as well as CD8 ^+^ T and NK cells. The expression of *CaMKⅡ* was positively correlated with that of *PDCD1* and *LAG3*. Correspondingly, the expression of *FAK* was negatively correlated with that of *TNFSF9*, *TNFRSF4*, and *TNFRSF18*. Collectively, extracellular calcium accelerates HepG2 cell proliferation and migration *via* FAK and CaMKⅡ and enhances cisplatin resistance. FAK and CaMKⅡ shape immune cell infiltration and responses in tumor microenvironments, thereby serving as potential targets for hepatoblastoma.

## Introduction

Hepatoblastoma is the most frequent primary liver malignancy in children younger than five years old, accounting for more than 80% of all pediatric cases with liver cancer ^[ 1– 2] ^. The incidence of hepatoblastoma is related with the Beckwith-Weidemann syndrome, familial adenomatosis polypi and low birth weight ^[ 3] ^. It was reported that the number of hepatoblastoma cases was rising by 2.2% each year between 2000 and 2015 ^[ 4] ^. Current treatment includes surgical resection combined with the platinum-based chemotherapy ^[ 5] ^. However, therapeutic effects of advanced tumors are still far from being satisfying. Multiple drug resistance is a major factor contributing to the unfavorable prognosis of children with an advanced-stage of hepatoblastoma ^[ 6] ^. There is an urgent need for investigations to focuse on factors responsible for drug resistance in hepatoblastoma and on possible ways to overcome the therapy resistance. The HepG2 cell line is a hepatoblastoma cell line ^[ 7] ^, and its genetic profile shows the presence of β-catenin mutation-related Wnt pathway activation ^[ 8] ^.

Calcium ions (Ca ^2+^) impact almost every aspect of cellular life ^[ 9] ^. In the resting state, the free calcium concentration in the cytosol is stable at approximately 100 nmol/L, which is called calcium homeostasis ^[ 9] ^. When cells are stimulated, cytosolic calcium concentration can increase more than 10-fold and locally up to 100- to 1 000-fold, mainly coming from outside of the cell and endoplasmic reticulum/sarcoplasmic reticulum ^[ 10] ^. The calcium signal is an important modulator of a spectrum of cellular processes, many of which are related to those important in cancer progression, such as proliferation and migration ^[ 11] ^. One concrete example of the role of calcium signaling in proliferation could be observed in early G1 of the cell cycle, where Ca ^2+^ was linked to the expression of the early response genes, such as *FOS*, *JUN*, and *MYC* ^[ 11] ^. Ca ^2+^ is also one of the important modulators of cell migration, and an influx of calcium ions is crucial for the migration of multiple cell types, including tumor cells ^[ 12] ^.

Intriguingly, when performing transfection experiments on HepG2 cells using a calcium phosphate method, we noticed an obviously altered morphology of the cells. We hypothesized that the addition of calcium ions could account for the morphology change. Therefore, we examined the changes in the expression of molecules associated with adhesion and metastasis by adding different concentrations of CaCl _2_ to the DMEM medium supplemented with 10% fetal bovine serum (FBS). To investigate the role of extracellular calcium in proliferative and metastatic behavior, we measured cell proliferation and migration in the CaCl _2_-treated HepG2 cells. We also explored the molecular mechanisms by quantifying the activity of intracellular signaling pathways, particularly the activation of focal adhesion kinase (FAK) and its regulatory phosphatase Ca2 ^+^/calmodulin-dependent kinase Ⅱ (CaMKⅡ). We further explored the impact of calcium on sensitivity to the treatment of cisplatin in HepG2 cells. Moreover, we evaluated the significance of FAK and CaMKⅡ in hepatoblastoma using public database.

## Materials and methods

### Cell lines and culture

The HepG2, MCF-7, Caco-2, and HUVEC cell lines were obtained from the Chinese Academy of Sciences (Shanghai, China). All cell lines were cultured in the DMEM medium (Cat. #11965092, Gibco, New York, USA) supplemented with 10% FBS (Cat. #10099-141, Gibco), 100 units/mL penicillin, and 100 μg/mL streptomycin (Cat. #15140-122, Gibco), and incubated at 37 ℃ in an incubator containing 5% CO _2_.

### Drugs and reagents

The inhibitors of FAK (PF573228; Cat. #SC1099, Beyotime, Shanghai, China), CaMKⅡ (KN93; Cat. #HY-15465, MedChemExpress, New Jersey, USA), calpeptin (MDL28170; Cat. #HY-18236, MedChemExpress), ATPase [(-)-blebbistatin; Cat. #SF9087, Beyotime], actin polymers (Cytochalasin B; Cat. #HY-16928, MedChemExpress), mitosis (Nocodazole; Cat. #S1765, Beyotime), and Rho-associated coiled kinase (ROCK; Y-27632; Cat. #SC0326, Beyotime) were used to determine the involved mechanical components ^[ 13] ^. Calcium chloride anhydrous (CaCl _2_; Cat. #C1016, Sigma, Shanghai, China), potassium chloride (KCl; Cat. #10016308, Sinapharm Chemical Reagent Co., Ltd., Shanghai, China), and magnesium chloride hexahydrate (Sinapharm Chemical Reagent Co., Ltd.) were used to treat HepG2 cells with different final concentrations.

### RNA extraction and quantitative real-time PCR

Total RNA was isolated using Trizol (Cat. #15596026, Invitrogen, CA, USA) and transcribed into cDNA using the reverse transcription kit (Cat. #R323, Vazyme, Nanjing, Jiangsu, China) following the manufacturers' protocols. The cDNA was amplified with the AceQ Universal SYBR qPCR Master Mix 2X kit (Cat. #Q511, Vazyme) using the Step One Plus Real-Time PCR System (Applied Biosystems, Waltham, MA, USA). Primers were synthesized in Invitrogen (Shanghai, China) and the sequences were available in ***Supplementary Table 1*** (available online).

### Western blotting

Total proteins were obtained using the RIPA lysis buffer (Cat. #P0013C, Beyotime). The protein concentration was quantified by the bicinchoninic acid (BCA) assay kit (Cat. #23227, Thermo Scientific, Rockford, IL, USA). Then, proteins were separated using 8% to 15% SDS polyacrylamide gel electrophoresis, then transferred to PVDF membrane, and blocked in 5% bovine serum albumin (Cat. #A1933, Sigma, Louis, MO, USA). The membrane was probed with specific primary antibodies followed by horseradish peroxidase conjugated antibody. Proteins were detected by addition of ECL substrate (Cat. #34580, Thermo Fisher Scientific). The antibodies are listed in ***Supplementary Table 2*** (available online).

### Cell counting kit-8 assay

The cell counting kit-8 (CCK8) assay was carried out in line with the manufacturer's i nstructions (Cat. #CK04, Dojindi Labs, Kumamoto, Japan). The absorbance at 450 nm was determined using a spectrophotometer (Labsystems, Sweden).

### EdU proliferation assay

The cell proliferation assay was conducted using the BeyoClick EdU Cell Proliferation Kit with Alexa Fluor 488 (Cat. #C0071S, Beyotime). The numbers of proliferative cells were counted by the fluorescence microscope (Olympus, Japan).

### Wound healing assay

Cells were seeded in 12-well plates at 95% confluence, and then cells were scratched with a pipette tip and washed with PBS to remove the floating cells. Wounded monolayers were photographed at 12-h intervals for 72 h.

### Flow cytometry analysis

HepG2 cells were harvested after treatment with or without CaCl _2_ for 24 h, and then incubated with human Fc receptor blocker (Clone QA19A30; Cat. #163404, BioLegend, San Diego, CA, USA) to prevent non-specific binding. The cells were then stained with PE/Cyanine7 anti-human CD274 (B7-H1, PD-L1) antibody (Clone 29E.2A3; Cat. #329718, BioLegend). The BD FACSCanto Ⅱ flow cytometer was used to perform flow cytometry, and FlowJo software (Tree Star, OR, USA) was used to analyze the data.

### Data processing

RNA-seq data and corresponding clinical information on hepatoblastoma were obtained from the GEO database (GSE104766, GSE131329, GSE133039, GSE151347, and GSE38122). All expression profiles were transformed into TPM (transcripts per million transcripts). Differential gene expression analysis was performed using the R 'limma' package ( https://bioconductor.org/packages/release/bioc/html/limma.html) in R. The Wilcoxon rank‐sum test was implemented to analyze the differences between different groups.

### Pathway analysis

Based on the transcriptome profiling data and gene sets retrieved from Molecular Signatures Database (MSigDB) ^[ 14] ^, the gene set enrichment analysis (GSEA) was performed to analyze the KEGG pathways and Hallmark gene sets between different groups *via* the R 'clusterprofiler' package ( https://bioconductor.org/packages/release/bioc/html/clusterProfiler.html). The protein-protein interaction (PPI) network diagrams of differentially expressed genes (DEGs) were drawn using the Cytoscape software ( https://cytoscape.org/).

### Tumor immune micro-environment analysis

The Microenvironment Cell Populations-counter (MCP-counter), CIBERSORT, and Xcell algorithms assessed the abundance of immune cells among different groups. The immune score, stroma score, and microenvironment score were computed using the R package 'Xcell' ( https://github.com/dviraran/xCell). Correlations among the expression levels of *CaMKⅡ* and *FAK* and measurments of different immune checkpoints were calculated using Spearman's correlation analysis.

### Statistical analysis

All data were presented as the mean ± standard deviation from three independent experiments. Statistical significance was performed using either one-way ANOVA with a Bonferroni post hoc test or Student's *t*-test. All statistical analyses were performed with GraphPad version 9.0 software (GraphPad Software, San Diego, CA, USA). For bioinformatics analysis, statistical analysis was performed by R (version 4.0.1, https://www.r-project.org/). Wilcoxon rank-sum test was used to compare the two groups. The Kruskal-Wallis test was applied to compare multiple groups. Spearman correlation analysis was used for correlation analysis. *P* < 0.05 was considered statistically significant.

## Results

### Calcium ions induced morphological changes and enhanced the epithelial-mesenchymal transition in HepG2 cells

**Figure 1:**
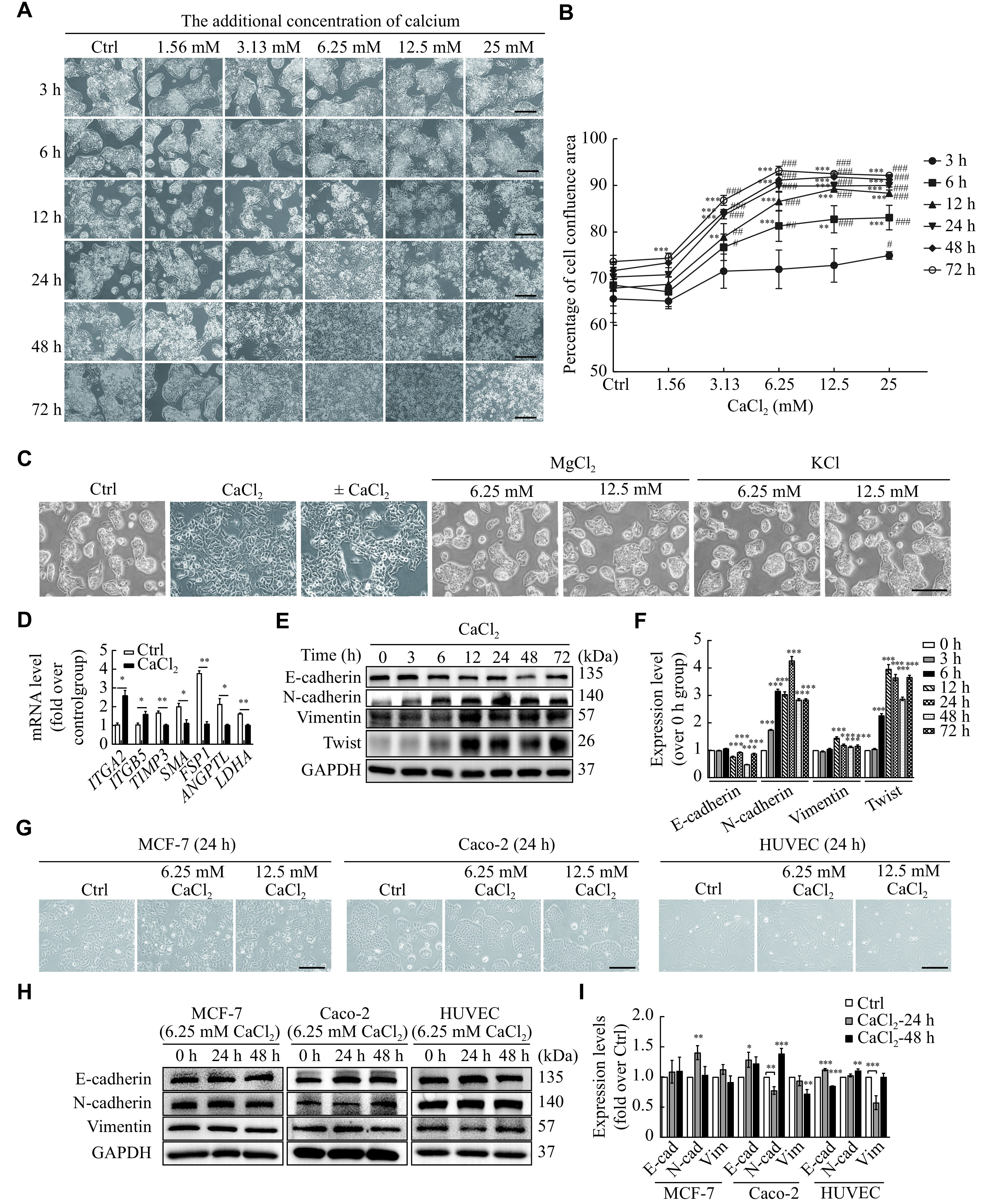
Calcium ions induced the morphological changes and enhanced the epithelial-mesenchymal transition in HepG2 cells.

To study the roles of extracellular calcium on HepG2 cells, we added additional different concentrations of CaCl _2_ to cells' regular medium (the DMEM medium supplemented with 10% FBS). As shown in ***Fig. 1A*** and ***1B***, HepG2 cells grew in the form of cell mass, then generated cell protrusions after the addition of CaCl _2_, and spread out with the increase of CaCl _2_ treatment time. In addition, morphological changes in HepG2 cells were more profound at higher concentrations of 6.25, 12.5, and 25 mmol/L CaCl _2_ than at 1.56 and 3.13 mmol/L CaCl _2_. Notably, the addition of MgCl _2_ and KCl could not induce the altered morphology of HepG2 cells ( ***Fig. 1C***). Moreover, when the extra addition of CaCl _2_ was removed, the changed morphology was reversed. Therefore, it was the calcium ions that changed the morphology of HepG2 cells ( ***Fig. 1C***).

As we all know, the epithelial-mesenchymal transition (EMT) plays a key role in the development of cell motility and invasiveness. We examined the mRNA expression levels of adhesion-related molecules by qRT-PCR. As shown in ***Fig. 1D***, CaCl _2_ increased *ITGA2* and *ITGB5* mRNA levels, but decreased *TIMP3*, *SMA*, *FSP1*, *ANGPTL*, and *LDHA* mRNA levels, compared with the control group. In addition, we investigated the ability of calcium ions to alter markers of EMT in HepG2 cells, and found that the addition of calcium ions reduced the epithelial marker E-cadherin from 12 to 72 h ( ***Fig. 1E*** and ***1F***), with the lowest level at 48 h. Meanwhile, the mesenchymal markers (N-cadherin and vimentin) and transcription factor Twist were significantly increased, compared with the control group ( ***Fig. 1E*** and ***1F***). These indicate that extracellular calcium ions may mediate HepG2 cell motility by initiating the EMT. Furthermore, we examined the effects of calcium ions on other tumor and normal cells. As a result, the addition of CaCl _2_ did not induce noticeable morphological alterations in MCF-7, Caco-2, and HUVEC cell lines ( ***Fig. 1G***). Correspondingly, it did not simultaneously reduce the epithelial marker E-cadherin and increase the mesenchymal markers (N-cadherin and vimentin) ( ***Fig. 1H*** and ***Fig. 1I***). The results indicate that extracellular calcium may alter HepG2 cell morphology by initiating the EMT.

### Calcium ions promoted HepG2 cell proliferation and migration

**Figure 2:**
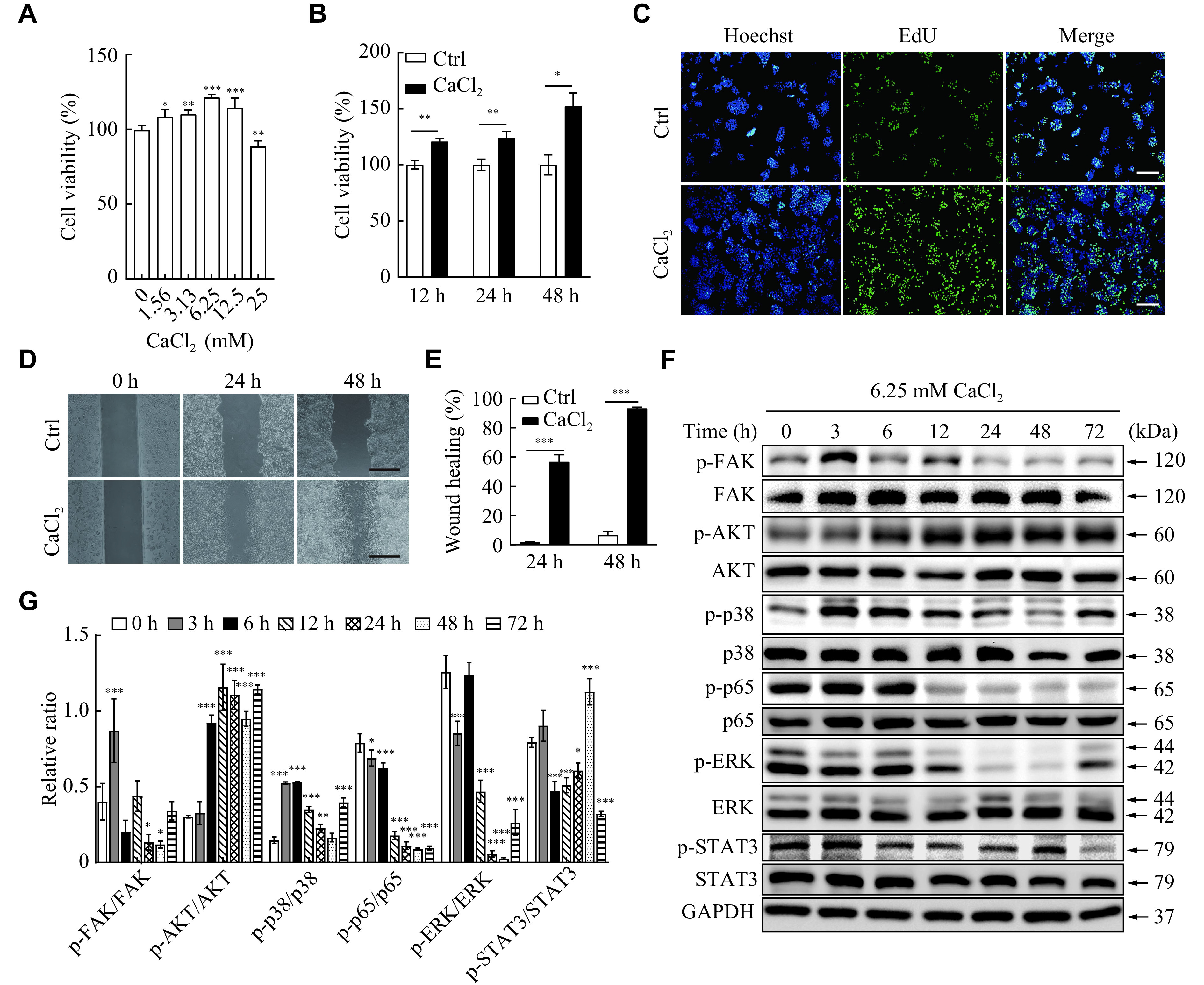
Calcium ions promoted HepG2 cell proliferation and migration and changed the phosphorylation levels of multiple proteins.

To determine whether extracellular calcium ions affect the malignant biological behavior of HepG2 cells, we analyzed the proliferation and migration of HepG2 cells in the presence or absence of CaCl _2_. CCK8 assays showed that CaCl _2_ dose-dependently promoted HepG2 cell viability, when the concentration of additional CaCl _2_ was between 0 and 6.25 mmol/L ( ***Fig. 2A***). When the CaCl _2_ concentration continued to increase, its effect on HepG2 began to decrease, showing a relative inhibitory effect at 25 mmol/L ( ***Fig. 2A***). According to these results, the concentration of 6.25 mmol/L CaCl _2_ was used for subsequent experiments. The CCK8 assay ( ***Fig. 2B***) showed that the proliferation of HepG2 cells was increased by the treatment of CaCl _2_. Moreover, the EdU assay was also used to detect the effect of CaCl _2_ on cell proliferation. As shown in ***Fig. 2C***, the number of EdU positive cells increased clearly after CaCl _2_ treatment for 24 h. The effect of CaCl _2_ on HepG2 cell migration was examined by using wound healing assays. As shown in ***Fig. 2D*** and ***2E***, CaCl _2_ could promote HepG2 cell migration, compared with the control group. Taken together, these results reveal that calcium ions can promote HepG2 cell proliferation within a certain range of concentrations and enhance HepG2 cell migration.

To investigate the signaling pathways involved in the CaCl _2_-mediated effect on HepG2 cell proliferation and migration, we detected the phosphorylation levels of FAK, protein kinase B (AKT), p38 mitogen-activated protein kinase, nuclear factor kappa-B (NF-κB) p65, extracellular signal-regulated kinase (ERK), and signal transducer and activator of transcription 3 (STAT3) by Western blotting. The data indicated that the phosphorylation level of FAK was obviously enhanced in response to CaCl _2_ treatment at 3 h and then decreased at 48 and 72 h ( ***Fig. 2F*** and ***2G***). Phosphorylation of AKT was significantly increased 6 h after the addition of CaCl _2_, and p38 phosphorylation was increased 3 h after treatment of CaCl _2_. Meanwhile, phosphorylation of p65 and ERK was impaired 12 h after CaCl _2_ treatment, and the phosphorylation level of STAT3 was decreased 6 to 24 h after the addition of CaCl _2_. Therefore, the results indicate that calcium ions may promote HepG2 cell proliferation and migration by regulating the phosphorylation levels of FAK, AKT, p38, p65, ERK, and STAT3.

### Calcium ions promoted HepG2 cell proliferation and migration by enhancing the activation of FAK-AKT signaling pathways

**Figure 3:**
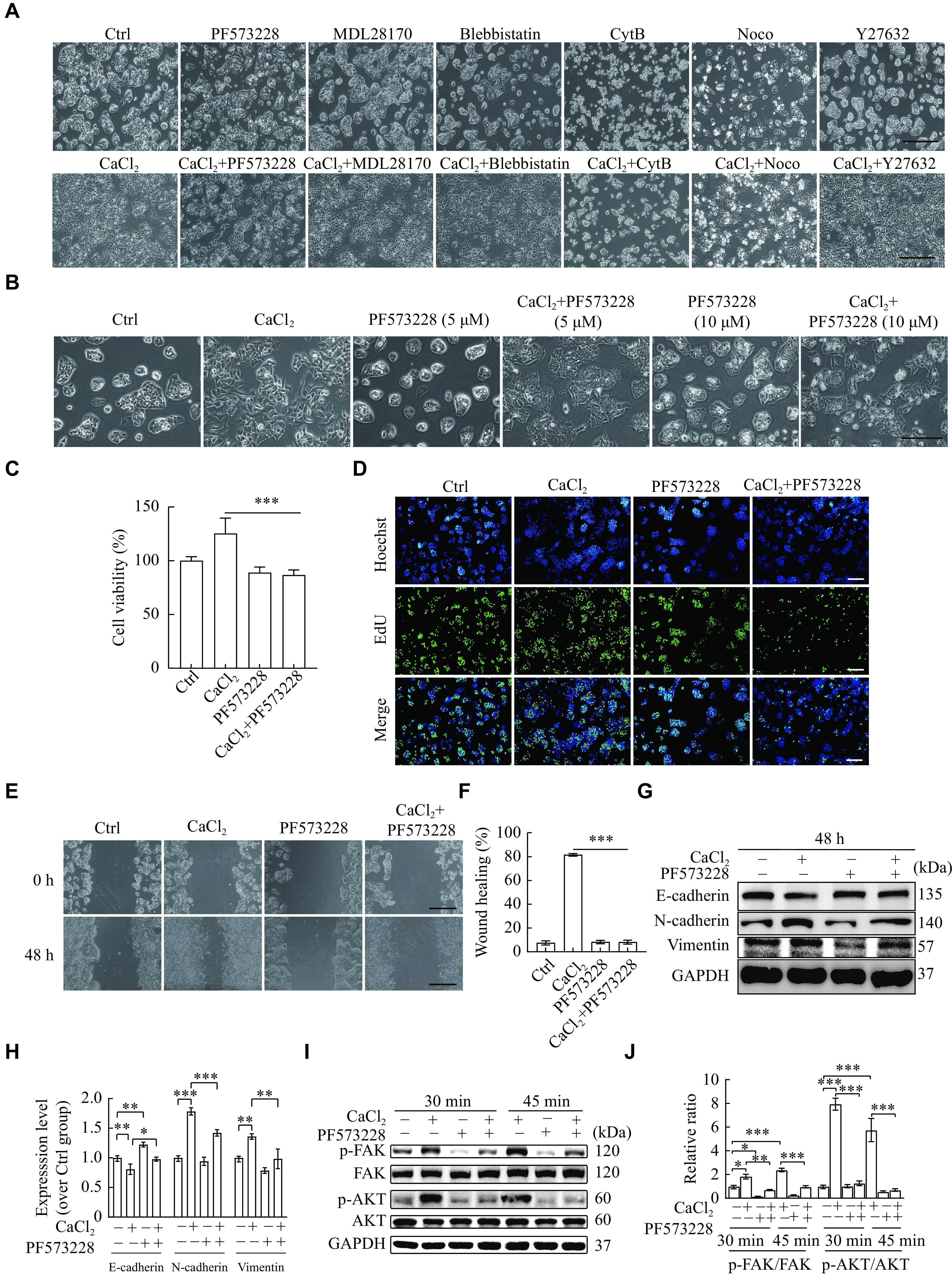
Calcium ions promoted HepG2 cell proliferation and migration by enhancing the activation of FAK-AKT signaling pathways.

The inhibitor of FAK (PF573228), calpeptin (MDL28170), ATPase [(-)-blebbistatin], actin polymers (cytochalasin B), mitosis (nocodazole), and ROCK (Y-27632) were used to explore possible mechanisms involved. We found that the changed morphology of HepG2 cells was reversed, when pretreated with PF573228 (10 µmol/L) for 1 h, and then stimulated with CaCl2 for 12 h ( ***Fig. 3A***). No similar inhibitory effects were observed, when the cells were pretreated with the other five compounds associated with cells mobility ( ***Fig. 3A***). These results are consistent with those of a previous study ^[ 15] ^. Next, two doses (5 and 10 µmol/L) of PF573228 were used to treat cells. As shown in ***Fig. 3B***, the effect of PF573228 was concentration-dependent, and a 5 µmol/L dose was used for subsequent experiments due its obvious inhibitory effects.

As shown in ***Fig. 3C***, PF573228 pretreatment clearly reduced HepG2 cell proliferation, compared with the cells treated with CaCl _2_. The same results were verified by the EdU assay ( ***Fig. 3D***). The wound healing assay showed that PF573228 reversed the promoted effects of CaCl _2_ on cell migration ( ***Fig. 3E*** and ***3F***). Moreover, we found that PF573228 pre-treatment reversed the CaCl _2_-induced changes in expression levels of EMT marker proteins ( ***Fig. 3G*** and ***3H***). At the same time, the addition of PF573228 clearly decreased the phosphorylation levels of FAK and AKT, compared with CaCl _2_ alone ( ***Fig. 3I*** and ***Fig. 3J***). Overall, the results suggest that calcium ions may promote HepG2 cell proliferation and migration by enhancing the activation of FAK-AKT signaling pathway.

### Calcium ions promoted HepG2 cells proliferation and migration *via* CaMKⅡ-FAK-AKT signaling pathway

**Figure 4:**
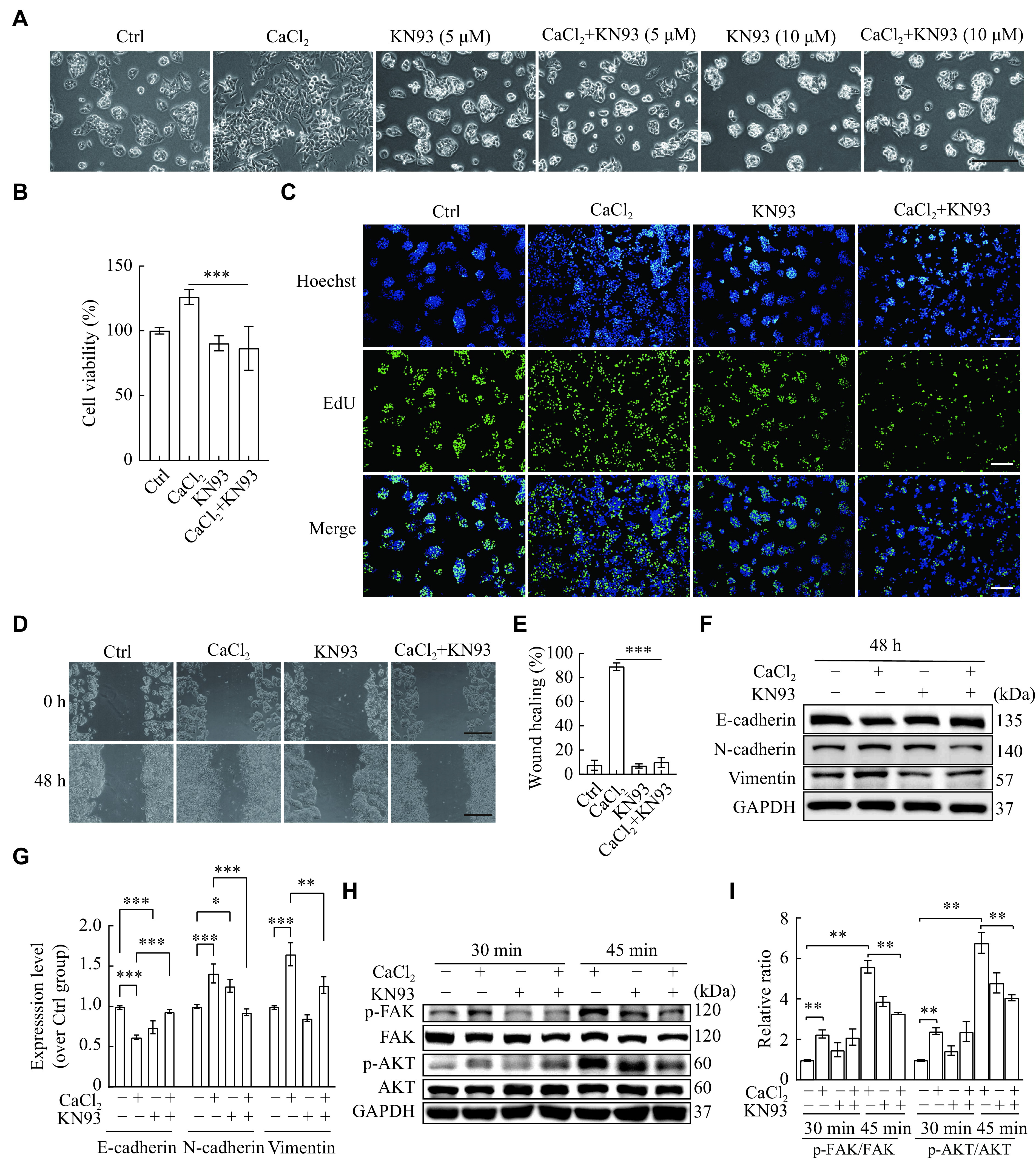
Calcium ions promoted HepG2 cell proliferation and migration *via* CaMKⅡ-FAK-AKT signaling pathway.

Next, the upstream factors regulating the phosphorylation of FAK were investigated. It has been described that CaMKⅡ regulates cell motility by stimulating tyrosine dephosphorylation of focal adhesion proteins to promote focal adhesion turnover ^[ 16] ^. Thus, CaMKⅡ may be an upstream factor that regulates the phosphorylation of FAK. Therefore, we used KN93, the inhibitor of CaMKⅡ, to pretreat HepG2 cells with different concentrations (5 and 10 μmol/L). The data showed that KN93 inhibited the effect of CaCl _2_ in a concentration-dependent manner ( ***Fig. 4A***), and 5 μmol/L of KN93 was chosen for the subsequent experiments due to its obvious reversal effect.

As shown in ***Fig. 4B***, cell proliferation abilities were reduced with KN93 pretreatment, compared without KN93 pretreatment. The same results were achieved with the EdU assay ( ***Fig. 4C***). Next, we analyzed the effect of KN93 on migration abilities of HepG2 cells by the wound healing assay. The migration of HepG2 cells was significantly inhibited in the presence of KN93, compared without KN93 pretreatment ( ***Fig. 4D*** and ***4E***). In addition, KN93 pretreatment reversed EMT induced by CaCl _2_ and reduced the phosphorylation levels of FAK and AKT, compared with CaCl _2_ treated alone ( ***Fig. 4F***– ***4I***). Collectively, these results indicate that CaMKⅡ is the upstream factor regulating the phosphorylation of FAK. Hence, calcium ions may promote HepG2 cell proliferation and migration *via* the CaMKⅡ-FAK-AKT signaling pathway.

### Calcium ions decreased HepG2 cells' sensitivity to cisplatin

**Figure 5:**
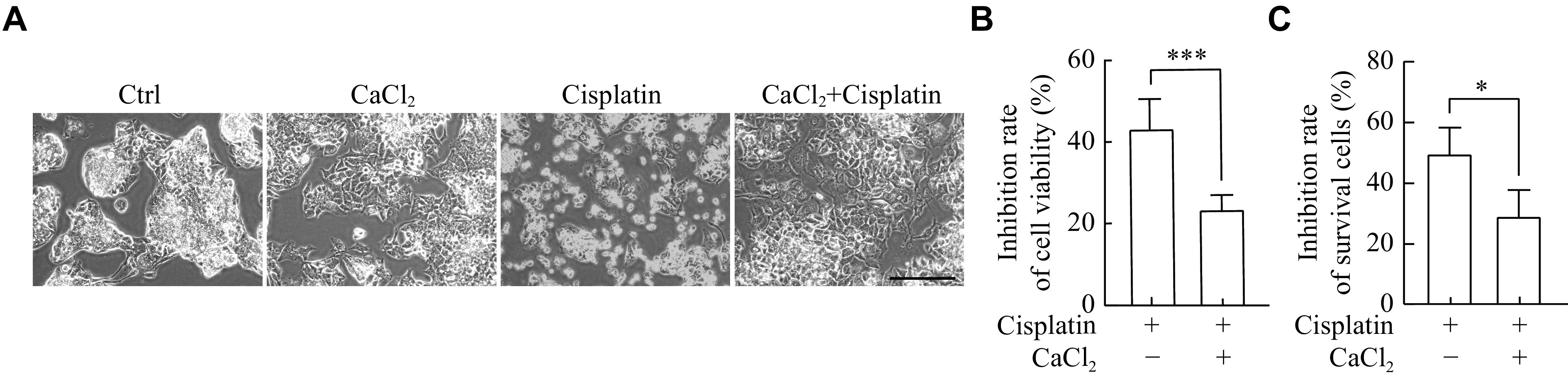
Calcium ions decreased the cisplatin sensitivity of HepG2 cells.

Next, we investigated the effect of CaCl _2_ on cisplatin-induced cell death. HepG2 cells pre-treated with CaCl _2_ showed an obviously reduced proportion with typical apoptotic features, such as rounding, shrinkage, and detachment ( ***Fig. 5A***). The quantification of cell viability by the CCK8 assay showed that the inhibition rate of cisplatin was reduced by 20.34 percentage points in the group of CaCl _2_ + cisplatin, compared with cisplatin alone ( ***Fig. 5B***). To further confirm the decrease in the inhibition rate, survival cells were counted. As shown in ***Fig. 5C***, the cells treated with cisplatin in the presence of CaCl _2_ showed a reduced inhibition rate (50.47% *vs.*29.34%), compared with the cisplatin group, suggesting that calcium ions decreased HepG2 cells' sensitivity to cisplatin.

### Characterization of FAK and CaMKⅡ expression in hepatoblastoma

**Figure 6:**
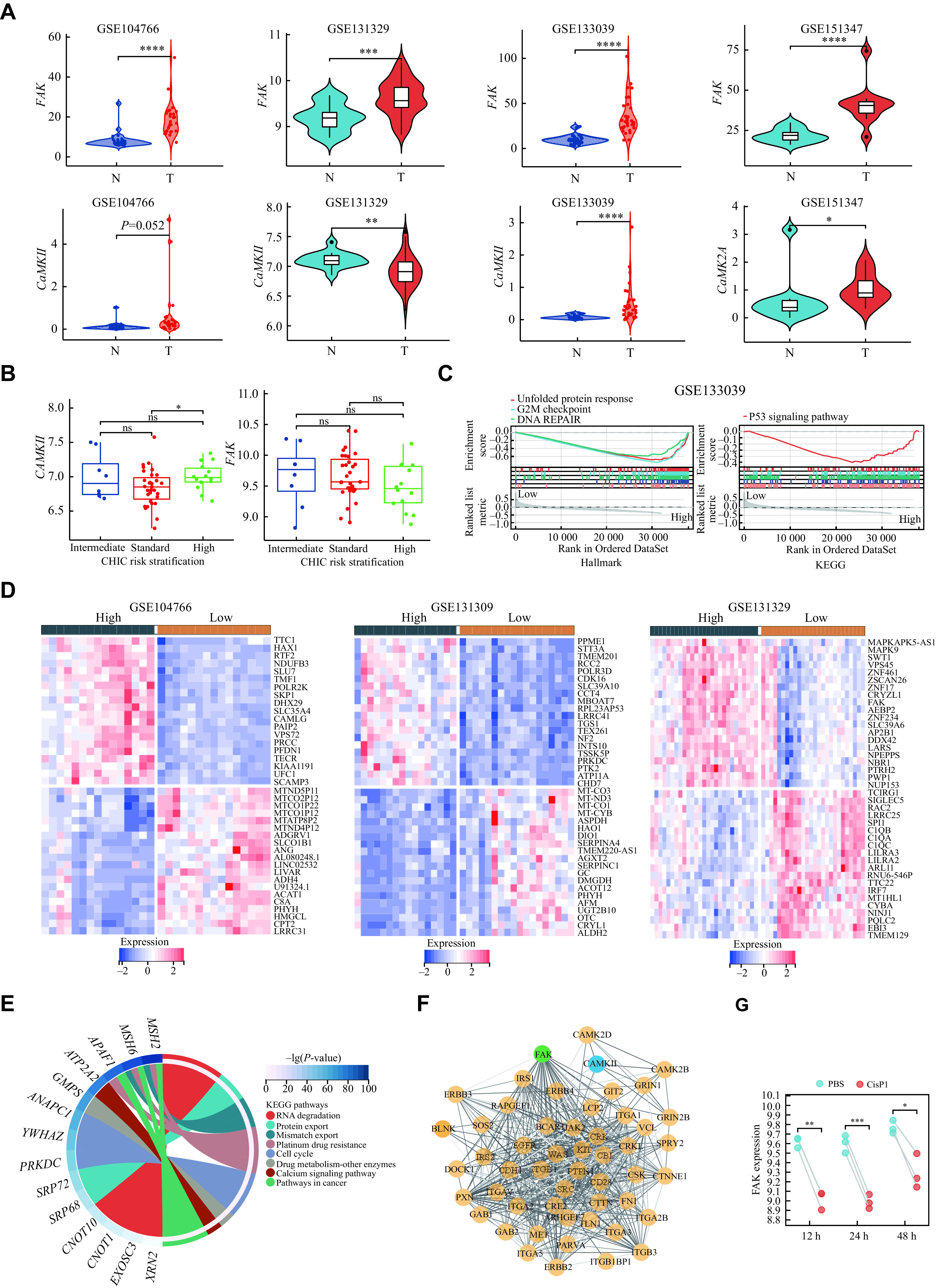
Characterization of FAK and CaMKⅡ expression in hepatoblastoma.

To investigate the roles of FAK and CaMKⅡ in hepatoblastoma, we further analyzed RNA‐seq data and corresponding clinical information on hepatoblastoma from the GEO database. As shown in ***Fig. 6A***, *FAK* mRNA expression level was significantly increased in hepatoblastoma. Similarly, *CaMKⅡ* was mostly increased in hepatoblastoma. Using Children's Hepatic tumor International Collaboration (CHIC) risk stratification system, we found that there was a significant difference of *CaMKⅡ* expression between the standard and high CHIC risk stratification groups from GSE133039 ( ***Fig. 6B***). Moreover, we combined the expression of *FAK* and *CaMKⅡ*, and categorized them as low and high groups. Intriguingly, GSEA enrichment analysis of hallmark gene sets in GSE133039 showed that the high expression of *FAK* and *CaMKⅡ* was enriched in unfolded protein response, G2M checkpoint, and DNA repair ( ***Fig. 6C***). The KEGG analysis indicated that p53 signaling pathway was enriched with high expression of *FAK* and *CaMKⅡ* ( ***Fig. 6C***). The differential expressed genes in three GEO datasets (GSE104766, GSE131309, and GSE131329) were further analyzed in groups with high and low expression of *FAK* and *CaMKⅡ*, and a total of 186 common genes were found ( ***Fig. 6D***, ***Supplementary Fig. 1*** [available online]). The enriched KEGG pathways were identified as RNA degradation, cell cycle, and protein export, *etc*. ( ***Fig. 6E***). The PPI network analysis of the 186 DEGs was further explored using the Cytoscape software ( ***Fig. 6F***), and the enriched KEGG and GO pathways were analyzed ( ***Supplementary Fig. 1B*** and ***1C ***). Intriguingly, the data from GSE38122 showed that *FAK* expression was significantly downregulated after cisplatin treatment on HepG2 cells ( ***Fig. 6G***), and no change of *CaMKⅡ* expression was observed ( ***Supplementary Fig. 1D ***).

### FAK and CaMKⅡ shaped immune cell infiltration and immune responses in hepatoblastoma

**Figure 7:**
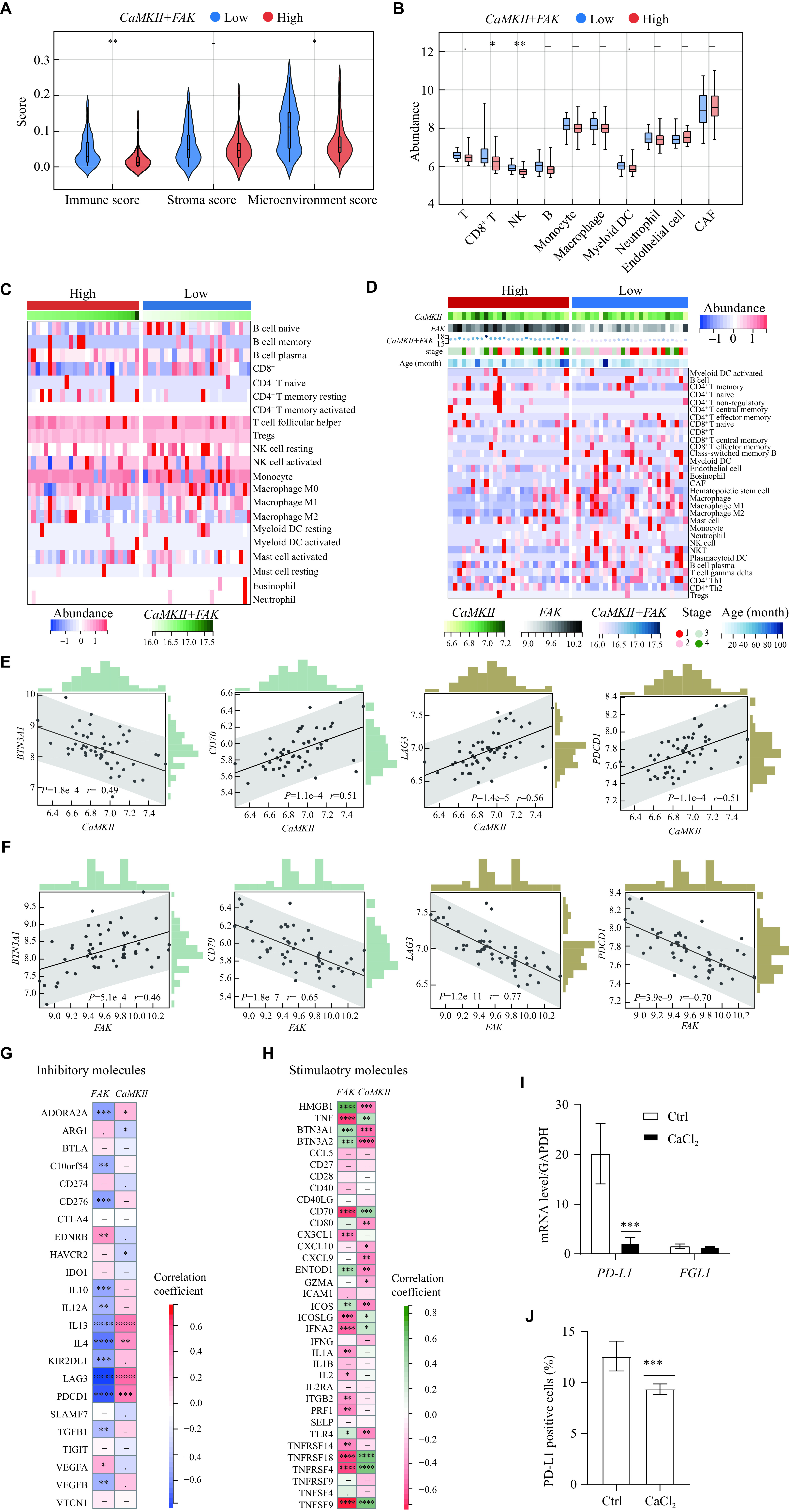
FAK and CaMKⅡ shaped immune cell infiltration and immune responses in hepatoblastoma.

Furthermore, we explored the roles of FAK and CaMKⅡ in tumor microenvironment of hepatoblastoma. There were significantly lower immune and microenvironment scores observed in the group with high expression of *FAK* and *CaMKⅡ* ( ***Fig. 7A***) as well as CD8 ^+^ T and NK cells ( ***Fig. 7B***). The immune cells infiltration was further analyzed by CIBERSORT ( ***Fig. 7C***) and Xcell algorithms ( ***Fig. 7D***). More immune cells were observed in the group with low expression of *FAK* and *CaMKⅡ* ( ***Fig. 7C*** and ***7D***). The expression of *CaMKⅡ* was positively correlated with that of *CD70*, *LAG3*, and *PDCD1* ( ***Fig. 7E***), while *FAK* exhibited negative correlations ( ***Fig. 7F***). Moreover, we analyzed the correlations of *FAK* and *CaMKⅡ* with a variety of inhibitory and stimulatory molecules ( ***Fig. 7G*** and ***7H***). Consequently, the expression of *CaMKⅡ* was positively correlated with that of *LAG3*, *PDCD1*, *IL-4*, and *IL-13* ( ***Fig. 7G***), but the expression of *FAK* was negatively correlated with that of *TNFSF9*, *TNFRSF4*, and *TNFRSF18* ( ***Fig. 7H***). Furthermore, we analyzed the expression of PD-L1 and FGL1 on HepG2 cells treated with or without CaCl _2_. The data showed that calcium treatment reduced PD-L1 expression at both mRNA and protein levels, compared with the control group ( ***Fig. 7I*** and ***7J***, ***Supplementary Fig. 2*** [available online]), while no difference in the expression of *FGL1* was observed at the mRNA level ( ***Fig. 7I***).

## Discussion

Ca ^2+^ is a critical regulator of various cancer processes ^[ 12, 17] ^. Many transmembrane Ca ^2+^ channels or pumps have abnormal expression or activities in tumor cells, facilitating oncogenesis and tumor progression ^[ 18– 19] ^. Previously, several studies have found that overexpression of transmembrane Ca ^2+^ channels increases Ca ^2+^ influx and stimulates intracellular Ca ^2+^-involved proliferative pathways ^[ 20– 21] ^. In the present study, extracellular calcium ions were shown to induce the morphology and adhesion molecule changes of HepG2 cells. However, similar changes were not found in other cancer cells, such as MCF-7, Caco-2, and HUVEC cells. It is possible that different cells respond differently to the treatment with extracellular calcium ions.

The microenvironment of both primary tumors and corresponding metastatic sites is a critical element in tumor progression ^[ 22] ^. Ca ^2+^ signal represents a potential means through which tumor microenvironment signals to cancer cells ^[ 23– 24] ^. The role of Ca ^2+^ signals in multiple cell types is still relatively unclear. Studies have demonstrated that Ca ^2+^ signal interacts with many microenvironmental factors. For example, the acidic characteristics of tumor microenvironment alters free Ca ^2+^ levels *via* the acid-sensing ion channel 1 (ASIC1), which causes the induction of reactive oxygen species and the activation of NF-κB in breast cancer cells ^[ 24] ^. In addition, a high level of extracellular Ca ^2+^ is a driver for bone metastasis in cancers, which expresses the extracellular calcium-sensing receptor (CaSR) ^[ 25] ^. Here, we demonstrated that extracellular calcium ions promoted HepG2 cell proliferation and migration within a suitable concentration range. High calcium ion concentration caused cell death, which may be related to cytosolic calcium overload ^[ 26] ^. Moreover, high calcium ion concentration can cause excessive mitochondrial calcium uptake, resulting in the release of cytochrome C and other pro-apoptotic factors ^[ 27] ^. A better understanding of both in intracellular and extracellular calcium ions may provide new insights into disease progression.

FAK, a non-receptor tyrosine kinase, was found to be activated and up-regulated in gliomas ^[ 28] ^. According to a previous study, the activated FAK could activate PI3K, and the activated PI3K would in turn activate AKT that regulated cell growth and movement ^[ 29] ^. In the present study, we consistently found that calcium ions increased the levels of p-FAK and p-AKT in HepG2 cells, and the inhibitor of FAK reduced the level of p-AKT.

Ca ^2+^/calmodulin (CaM)-dependent kinases belong to a family of multifunctional serine/threonine kinases regulated by the Ca ^2+^/CaM complex ^[ 30] ^. A recent study showed that the CaMKⅡ-regulated signaling also played a role in carcinogenesis ^[ 31] ^, and CaMKⅡ could phosphorylate several proteins involved in migration and invasion, including FAK ^[ 32] ^. Our results showed that the KN-93-mediated pharmacological inhibition of CaMKⅡ activity decreased the phosphorylation levels of FAK and AKT. Taken together, these findings indicate that the activation of the CaMKⅡ/FAK/AKT signaling axis plays an important role in the effects of calcium ions on HepG2 cell proliferation and migration. In addition, as the level of p-p38 was up-regulated and the levels of p-p65, p-ERK, and p-STAT3 were down-regulated, calcium ions may also regulate these signaling pathways to promote HepG2 cell growth, but these phenomina need to be clarified by further studies.

Furthermore, we compressively analyzed the roles of FAK and CaMKⅡ in hepatoblastoma, because their expression levels were significantly increased in hepatoblastoma. In addition, we found that FAK and CaMKⅡ shaped immune cell infiltration and immune responses in hepatoblastoma. Moreover, *CaMKⅡ* expression was positively correlated with some inhibitory molecules, but *FAK* expression was negatively correlated with some stimulatory molecules. Together, these suggest that FAK and CaMKⅡ may play critical roles in regulating tumor immune microenvironment. The influx and efflux of Ca ^2+^ are controlled by a diverse array of calcium channels and pumps as well as exchangers present on the plasma membrane and membranes of intracellular organelles ^[ 23] ^. The limitation of the present study is that we did not determine which calcium channel was involved in the effect of calcium ions on HepG2 cells. The blockers of calcium transporters warrant further investigations. Moreover, chelation therapy may need to be explored for hepatoblastoma treatment or immunotherapy.

Cisplatin is frequently applied as the first-line chemotherapeutic agent in the clinic against a wide spectrum of cancers ^[ 33] ^. It may exhibit cytotoxic properties and induce apoptosis by binding to DNA. However, cisplatin resistance has become one of the major medical problems for its valid treatment against malignancies ^[ 34] ^. It is unclear whether calcium ions may cause changes in cisplatin resistance in HepG2 cells, but we showed that calcium ions increased cisplatin resistance of HepG2 cells, which provides new insights into antitumor therapy.

Taken together, we found that the elevated calcium ions altered the morphology and expression level of adhesion molecules in HepG2 cells. Mechanistically, calcium ions promoted HepG2 proliferation and migration mainly by activating the CaMKⅡ-FAK-AKT signaling pathway. Moreover, FAK and CaMKⅡ shaped tumor immune environment by influencing immune cells and immune checkpoints. Calcium ions could also increase cisplatin resistance in HepG2 cells. Collectively, targeting calcium signaling and FAK and CaMKⅡ molecules may serve as new therapeutic tools to treat hepatoblastoma.
